# 12-week concurrent brisk walking and Taijiquan (Tai Chi) improve balance, flexibility, and muscular strength of Chinese older women

**DOI:** 10.1371/journal.pone.0293483

**Published:** 2023-10-26

**Authors:** Xiaorong Bai, Wensheng Xiao, Kim Geok Soh, Agudamu, Yang Zhang

**Affiliations:** 1 School of Physical Education, Huzhou University, Huzhou, China; 2 Department of Sports Studies, Faculty of Educational Studies, Universiti Putra Malaysia, Serdang, Malaysia; 3 Graduate School of Social Welfare, Sungkyunkwan University, Seoul, South Korea; 4 Institute of Sports and Health Industry, HEHA CAT Fitness, Changsha, China; 5 Independent person, Windermere, Florida, United States of America; Università degli Studi di Milano: Universita degli Studi di Milano, ITALY

## Abstract

Healthy aging is a global goal to enhance the quality of life for older persons. This study evaluated the benefits of 12-week concurrent brisk walking and Taijiquan. Healthy Chinese women aged 60 years and above were enrolled to the control (n = 26) and intervention (n = 25) groups. Participants in the intervention group engaged in three exercise sessions per week for 12 weeks, whereas control group engaged in free-living activities. Each exercise session consisted of 20–45 minutes of walking and 20–45 minutes of Yang style 24-form Taijiquan. 12-week exercise improved (*p* < 0.05) the sit and reach test (within-group mean difference: +5.6 cm; Hedges’ *g* = 0.77), handgrip strength (mean difference: +3.1 kg; *g* = 0.89), arm curl (mean difference: +2.1 repetitions; *g* = 0.69), chair stand (mean difference: +2.6 repetitions; *g* = 0.63), and one-legged standing (mean difference: +2.2 seconds; *g* = 1.07). There was no improvement in the circulatory health, body composition, or life satisfaction. Therefore, this concurrent brisk walking and Taijiquan training, which targets major whole-body muscle groups, could improve aging-critical flexibility, muscular fitness, and balance in older women. The exercise meets the current WHO guideline, is safe to perform, and could be campaigned as a health promotion for older persons.

## Introduction

As global life expectancy rises, costs of medical care associated with aging are emerging as a growing burden on global health [1]. The trend has prompted global governments and international societies to address the socioeconomic trajectory of an aging society. The Chinese government, for instance, has outlined the “Healthy China 2030” plan to guide the interventions aimed at its ongoing demographic shift. Among various strategies, regular exercise has been widely acknowledged as an alternative “medicine” for promoting the health of individuals of all ages [2].

For older persons, exercise has been shown to benefit multiple facets of physical and mental health [3]. Aging is a known cause of compromised circulatory health, characterized by diminished abilities in regulating blood pressure [4], cardiovascular capacity [5], and pulmonary function [6]. Given that these age-related declines in circulatory health are associated with premature mortality [7], exercise is recommended as an important preventive measure [8]. In the context of aging, anthropometric changes manifest as a decline in lean body mass and a concurrent rise in body fat [9], which are associated with several noncommunicable diseases. For instance, high waist circumference is an accurate indicator of hypertension, diabetes, cardiovascular disease, and all-cause mortality in older persons [10]. In general, physical activity has a positive effect on body composition [11], which in turn serves as a preventative measure against noncommunicable diseases. Meanwhile, the aging process is characterized by a reduction in body flexibility and, as a result, a decrease in the functional capacity required for daily activities [12], which could constrain later-life independence. As a result, flexibility is one of the essential components of physical health that older persons require, and engaging in exercise has the potential to postpone the onset of this reduction in functionality. In addition, subjective well-being is a major determinant of healthy aging in older persons and in this regard, physical activity is an independent predictor of well-being [13]. Based on strong evidence from multiple regions and age groups, the World Health Organization recommends older persons “should do varied multicomponent physical activity that emphasises functional balance and strength training at moderate or greater intensity on 3 or more days a week” [8].

Taijiquan is a traditional Chinese conditioning practice that is now a UNESCO Intangible Cultural Heritage. Taijiquan originated from military exercise, which includes a variety of competing movements, such as the thrust palm, the hammer strike, the arm swing, the crouch stance, the bow stance, and the empty stance. As a result, original Taijiquan movements are extremely aggressive in combat. As time and values change, contemporary Taijiquan becomes a health oriented conditioning practice. The development of this traditional conditioning exercise for mass practice has been managed by the State Physical Culture and Sport Commission from 1952. As a result, a number of different Taijiquan routines have been streamlined into standardized forms, namely the 24-form, 42-form, 48-form, and 88-form Taijiquan. Among these, the Yang style 24-form has gained widespread popularity for mass practice throughout China. Taijiquan has also been disseminated to Western nations, where its practice has been further simplified to fit in public health promotion efforts [14]. Current evidence suggests that Taijiquan can enhance cardiorespiratory function, immune function, mental control, flexibility, balance control, and muscle strength in older persons [15]. In addition, regular practitioners of Taijiquan demonstrate enhanced postural stability [16] and cognitive function [17].

Globally, physical activity levels have been steadily declining, and physical inactivity increases with age and is more prevalent among women [18]. In China, walking is the most common type of daily physical activity. Correspondingly, the Chinese government invests in city greenways infrastructure, a unique urban touch with Chinese characteristics, to promote physical activity [19]. Not only is brisk walking along city greenways accessible and safe, but it also reduces risks of noncommunicable diseases and falls through multiple mechanisms [20].

There are a substantial body of literature examining the advantages of either Taijiquan practice or walking as forms of exercise in promoting healthy aging [21, 22]. To our knowledge, however, there is a lack of research on the specific combination of these two conditioning practices. Nonetheless, we have two compelling justifications for investigating the concurrent implementation of brisk walking and Taijiquan. First, exercise should target four aspects of physical fitness, namely endurance, strength, flexibility, and balance, and these types can be combined for more comprehensive health benefits [23]. Not only does exercise involving multiple activities have a synergistic effect on health, but workouts that combine focused learning and relaxed mood may also improve the exercise adherence of older persons [24]. When prescribing exercise as a medicine, no single form of exercise can protect people from all potential health risks and people of different ages should engage in differentiated forms of exercise to improve their age-specific health objectives. Notwithstanding benefits from concurrent endurance and strength exercise for older persons are well documented [25], other mixed types of exercise programs, such as concurrent exercise aimed at enhancing both balance and flexibility, are relatively less explored [26]. Thus, it is necessary to investigate the effects of combining different forms of exercise to reduce noncommunicable diseases, accidental falls, and poor mental health in older persons.

Second, our recent meta-analysis has yielded compelling evidence illustrating the molecular mechanism on how exercise training could delay aging [27]. Notably, one specific advice that emerged from our findings is the adoption of whole-body exercise. In brief, it has been seen that engaging in lower-body exercises such as running, as well as upper-body exercises like resistance training, can up-regulate nicotinamide phosphoribosyltransferase, a key rate limiting enzyme for nicotinamide adenine dinucleotide biosynthesis, which is associated with a mirage of lifestyle diseases in aging. To optimize this enzyme expression in the skeletal muscles, it is necessary to train as many muscle groups as possible. Hence, we recommend older persons to engage in whole-body training, which may enhance whole-body nicotinamide phosphoribosyltransferase expression. In this regard, Taijiquan is a slow-motion type of exercise that improves flexibility, strength, and balance, and brisk walking is a moderate-motion type of lower-body exercise that improves endurance and strength. The integration of these two physical activities may hold slow aging promise for older persons.

Given the global trend of physical inactivity, the international community is continuously optimizing exercise programs that are effective and have a high rate of adherence among populations that have traditionally been inactive. This study developed a cultural exercise program with a focus on promoting exercise among older women. Therefore, the purpose of this study was to assess the effects of a concurrent brisk walking and Taijiquan exercise program on the health of Chinese older women.

## Methods

### Research design

**Fig 1:**
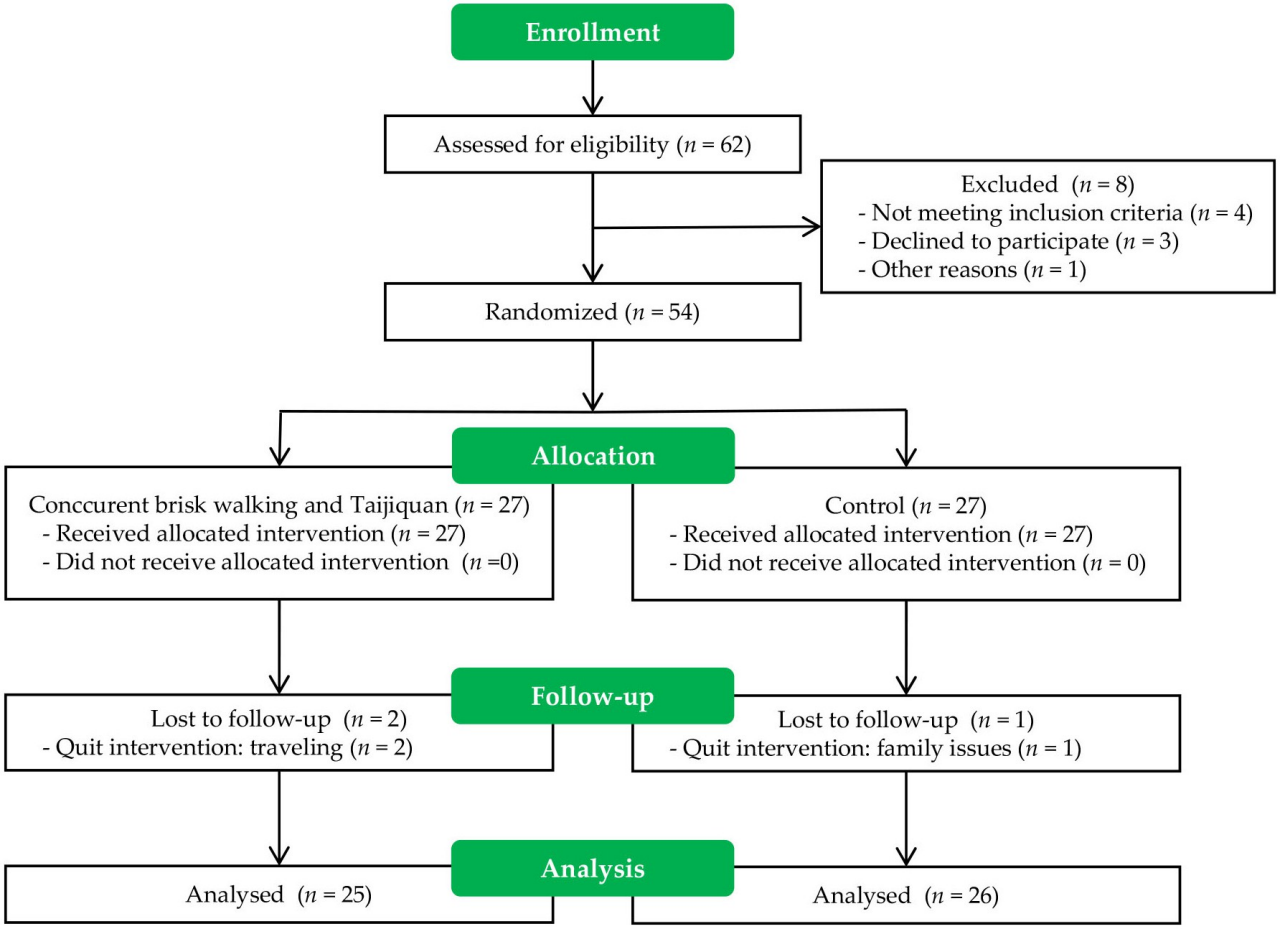
Cluster randomization procedure.

The research design was based on a cluster randomized controlled trial. Two senior institutions in Puyang, Henan province, were reached out to. For randomization, site administrators wrote the titles of the two institutions on paper and then placed them in two envelopes at random. Following the reordering of the envelopes, the principal investigator proceeded to select one of them, resulting in the inclusion of the corresponding institution in the control group, while the other institution was subsequently assigned to the intervention group. Statisticians were blinded to the group assignment. Fig 1 provides a summary of the study’s recruitment procedure. The sample size for a two-group experiment was determined using a *priori* power analysis. Based on an effect size of 0.19, type I error of 0.05, power of 0.80, and a possible 11% dropout rate in gerontology research [28], the total sample size was determined to be 54. During the follow-up period, a total of three participants were lost due to personal reasons. Consequently, the statistical analysis used a final sample size of 51.

This study was approved by the Universiti Putra Malaysia Ethics Committee (protocol code JKEUPM 2020–296). All participants gave a written informed consent, before formally enrolling in the research. Women aged 60 to 69 were invited to participate in the research. During the initial orientation, physicians were present to help identify potential risks of participation for each attendee. The criteria for exclusion were as follows: persons who performed less than 30 minutes of moderate-intensity physical activity per week; persons who were joining routine physical activity programs and were unable to adhere to the experimental protocol; persons who had a recent medical history (e.g., surgery within the past year); persons who had known cardiovascular and/or metabolic conditions (e.g., arrhythmia, hypertension, pre-diabetes); and, persons who had recently undergone surgical procedures on the knee, elbow, or shoulder, had a medical background of rheumatoid illness or neurological disease, and were currently undergoing treatment. Eligible participants were assigned to the control group and the brisk walking and Taijiquan group. Table 1 provides a summary of the demographic data. There were no statistically significant differences seen in the demographic characteristics at the baseline.

**Table 1:** Demographic characteristics.

Variables	C (*n* = 26)	BW+T (*n* = 25)	*p*
Age (year)	63.9 (2.9)	64.0 (2.6)	0.55
Height (cm)	157.8 (4.6)	157.6 (4.4)	0.84
Weight (kg)	65.8 (5.3)	65.7 (6.5)	0.90

Note. Data are expressed as mean (SD). BW+T, concurrent brisk walking and Taijiquan group; C, control group.

### Exercise program

**Fig 2:**
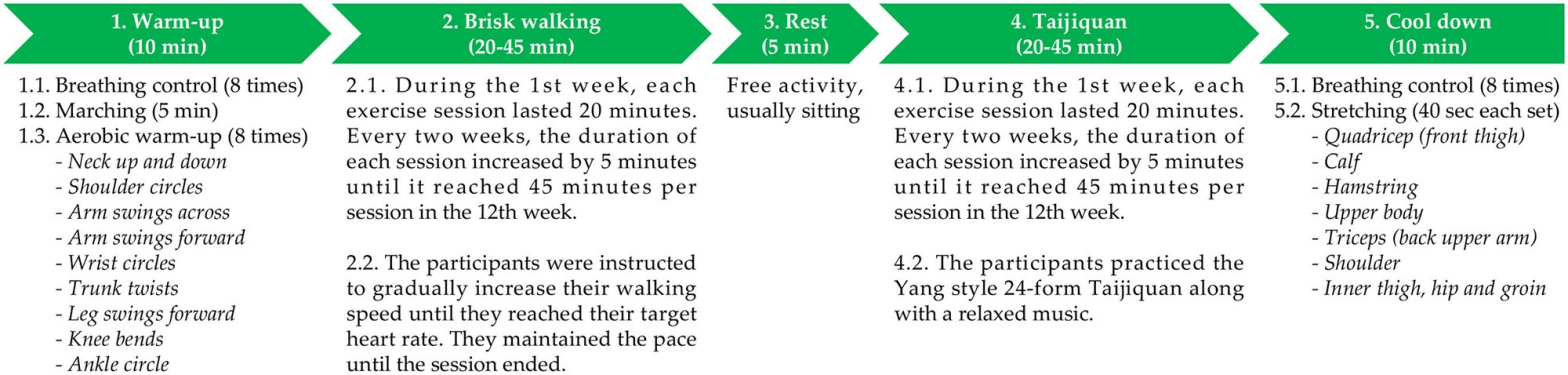
Study protocol.

**Fig 3:**
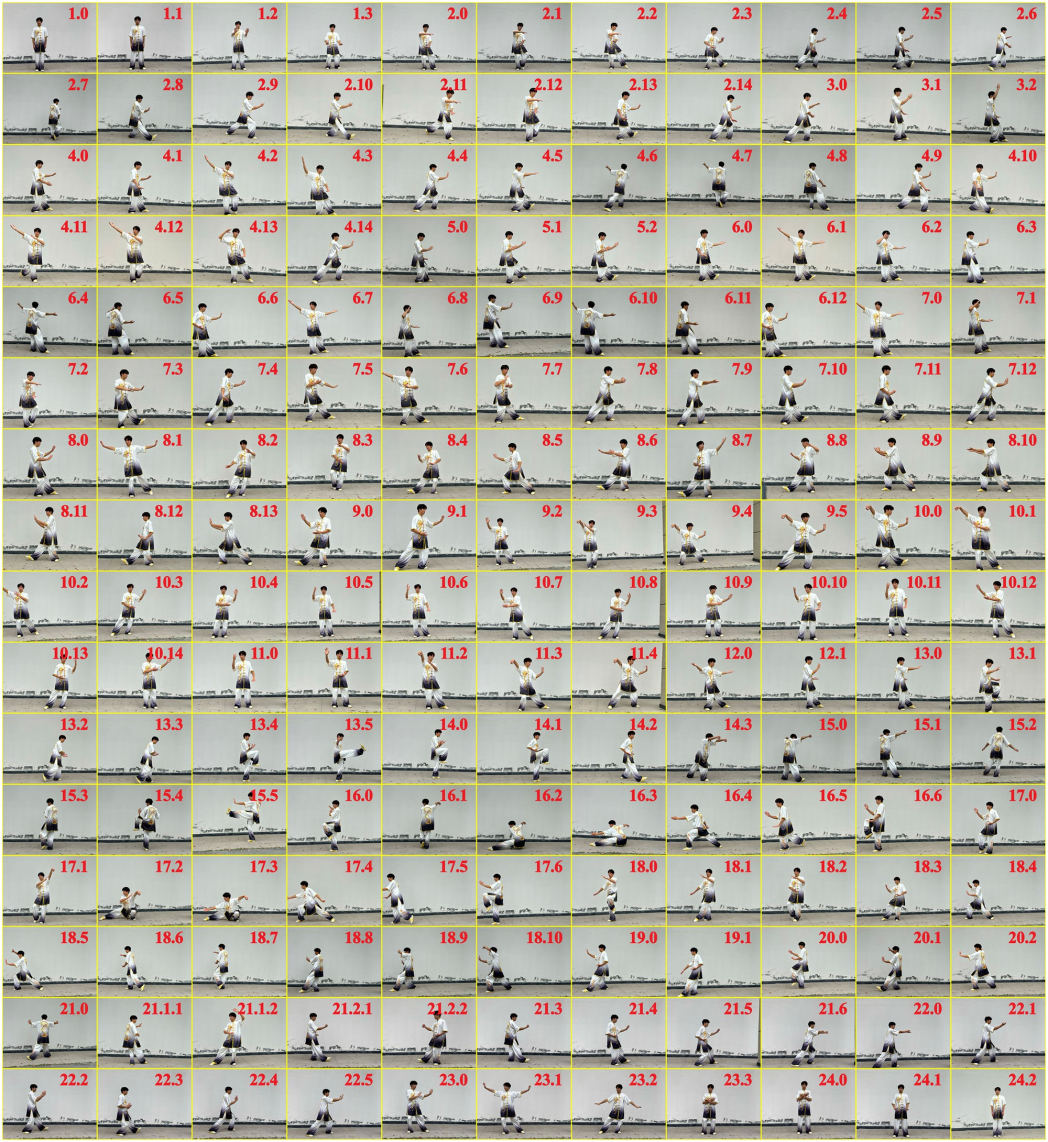
Decomposed movements of the Yang style 24-form Taijiquan. Readers are directed to go to the online edition for a high-quality image (DOI: https://doi.org/10.6084/m9.figshare.22336816.v1).

Exercises were conducted in Pushui Park, Puyang, Henan province. The intervention lasted for 12 weeks, during which the control group only engaged in daily living activities and the brisk walking and Taijiquan group engaged in structured exercise three times per week. Fig 2 is a summary of the exercise program. Given that the exercise intensity for the brisk walking segment targeted at the age predicted maximal heart rate, researchers instructed participants on how to use the smartwatch to monitor their real-time heart rate. During the familiarization phase, the intervention group was taught Yang style 24-form Taijiquan. Each movement of the Yang style 24-form Taijiquan is depicted in Fig 3. However, participants did not need to be proficient in Taijiquan to participate because instructors led each Taijiquan session.

### Outcome evaluation

Two days before the intervention and two days after the last exercise session, physical health and well-being were assessed. Participants were instructed to avoid strenuous physical activity and get adequate rest 24 hours before the evaluation. The physical examination included blood pressure, resting heart rate, vital capacity, waist circumference, hip circumference, waist-hip ratio, sit-and-reach test, back scratch, handgrip strength, arm curl, 30-second chair stand, and one-legged standing with the eyes closed. The measurement procedure was conducted in accordance with the National Physical Fitness Measurement Standards Manual (Elderly Version) [29]. During testing days, all equipment was calibrated on-site, and testers were competent at administering these measurements. As there is no official order for measurements, participants’ resting state-related components were assessed first, followed by other measurements in a convenient order. Well-being was examined by the Chinese-language satisfaction with life scale [30]. The scale consists of five statements and each statements was scored using a seven-point Likert scale. Total scores were between 5 to 35, with higher scores indicating better life satisfaction.

### Statistics

The de-identified data that support the conclusions of this study are available on figshare (DOI: https://doi.org/10.6084/m9.figshare.24117183.v1). Data were analyzed using the IBM SPSS version 23. Data were checked for normality and homogeneity of variances first. A generalized estimating equation model was used to examine the between-group difference. The statistical significance was determined using an alpha level of 0.05. The training effect is interpreted according to the gerontology-specific guideline [31] to accommodate data derived from the older population. The thresholds for small, medium, and large effects based on the Hedges’ *g* are 0.16, 0.38, and 0.76, respectively.

## Results

During the course of the study, no instances of injury were seen as a result of the exercise training program. Table 2 shows the evaluation results. At baseline, none of the physical health and well-being indicators were different between the two groups. After 12 weeks, there was a main time effect. While none of the indicators were changed in the control group, 12-week brisk walking and Taijiquan significantly (*p* < 0.05) improved the systolic blood pressure, diastolic blood pressure, resting heart rate, forced vital capacity, sit and reach test, back scratch, handgrip strength, arm curl, chair stand, one-legged standing, and life satisfaction. Meanwhile, 12-week brisk walking and Taijiquan did not affect waist circumference, hip circumference, and waist-hip ratio. Overall, 12-week brisk walking and Taijiquan had a large training effect on sit and reach test, handgrip strength, one-legged standing, and life satisfaction; a medium training effect on forced vital capacity, arm curl, and chair stand; and, a small training effect on systolic blood pressure, diastolic blood pressure, resting heart rate, and back scratch.

**Table 2:** 12-weeks exercise on health metrics.

Test battery	Time	Measurement		Between-group		Within-group *g*	
		C	BW+T	*p*	*g*	C	BW+T
**Circulatory health**							
***SBP (mm Hg)***	T0	125.0 (6.8)	125.1 (7.1)	0.96	0.01	0.11	0.32
	T12	125.7 (6.3)	122.8 (7.3)*	0.12	0.43		
***DBP (mm Hg)***	T0	74.4 (8.0)	75.3 (9.6)	0.74	0.10	0.01	0.16
	T12	74.4 (7.3)	73.8 (8.9)*	0.78	0.08		
***RHR (bpm)***	T0	73.7 (8.4)	74.0 (7.3)	0.90	0.03	0.09	0.34
	T12	74.4 (7.9)	71.5 (7.5)*	0.18	0.38		
***FVC (mL)***	T0	2316 (475)	2348 (377)	0.79	0.08	0.03	0.46
	T12	2302 (419)	2524 (408)*	0.05	0.52		
**Body composition**							
***WC (cm)***	T0	93.0 (6.8)	93.3 (6.9)	0.90	0.04	0.01	0.07
	T12	93.1 (6.9)	92.8 (7.1)	0.88	0.04		
***HC (cm)***	T0	102.2 (4.3)	102.3 (6.6)	0.95	0.02	0.01	0.09
	T12	102.2 (4.3)	101.7 (6.2)	0.76	0.09		
***WHR***	T0	0.9 (0.0)	0.9 (0.0)	0.83	0.01	0.01	0.01
	T12	0.9 (0.0)	0.9 (0.0)	0.86	0.01		
**Flexibility**							
***SR (cm)***	T0	3.8 (6.5)	3.8 (7.7)	0.99	0.01	0.03	0.77
	T12	4.0 (6.8)	9.4 (6.9)*#	0.01	0.79		
***BC (cm)***	T0	-6.9 (9.5)	-7.3 (8.6)	0.86	0.04	0.01	0.21
	T12	-6.8 (9.1)	-5.5 (8.2)*	0.60	0.15		
**Muscular fitness**							
***HGS (kg)***	T0	22.2 (3.6)	21.8 (3.6)	0.75	0.11	0.06	0.89
	T12	22.4 (3.0)	24.9 (3.4)*#	0.01	0.78		
***AC (rep)***	T0	19.7 (3.6)	19.7 (3.2)	0.99	0.01	0.03	0.69
	T12	19.6 (3.1)	21.8 (2.9)*#	0.01	0.67		
***CS (rep)***	T0	17.6 (3.5)	18.2 (4.2)	0.56	0.16	0.12	0.63
	T12	18.0 (3.1)	20.8 (4.0)*#	0.01	0.78		
**Balance (sec)**	T0	3.7 (2.1)	3.7 (2.0)	0.99	0.01	0.05	1.07
	T12	3.8 (2.3)	5.9 (2.1)*#	0.00	0.95		
**Life satisfaction**	T0	27.7 (3.6)	27.8 (3.0)	0.92	0.03	0.22	0.88
	T12	28.6 (4.4)	30.2 (2.4)*	0.74	0.45		

Note. Data are expressed as mean (SD). AC, arm curl; BS, back scratch, BW+T, concurrent brisk walking and Taijiquan group; C, control group; CS, chair stand; DBP, diastolic blood pressure; FVC, forced vital capacity; HC, hip circumferences; HGS, handgrip strength; RHR, resting heart rate; SBP, systolic blood pressure; SR, sit-and-reach test; T0, pre-intervention test; T12, 12-week post-intervention test; WC, waist circumferences; WHR, waist-to-hip ratio.

*Within-group T0 vs. T12, *p* < 0.05.

^#^ Between-group, *p* < 0.05.

## Discussion

We show that 12-week concurrent brisk walking and Taijiquan improved the flexibility, muscular fitness, and balance of Chinese women aged 60 and older. This study’s contribution not only compensates for the lack of research on the combination of balance and flexibility exercise program, but it also introduces a low risk of injury, equipment-free, convenient combination of exercises for older persons.

The improvement of upper-body muscular fitness is the most notable finding of this study. Although the intervention appears to have little to do with upper-body strength development when viewed through the lens of Western resistance training theory, which focuses on loads, repetitions, and volume [32], current intervention increased handgrip strength by 3.1 kg, which should be attributed solely to Taijiquan. The mechanism has Chinese cultural roots. Taijiquan was created as a form of combat skill, and it was never intended to be a gentle form of exercise, even though its modern motion appears slow and graceful during practice. As a result, its practitioners are expected to develop whole-body strength even for recreational purposes [33], and its combat power is augmented by a type of penetrating hand strike [34]. Neither is the present finding isolated. Barbat-Artigas et al. [35] examined a group of postmenopausal women (mean age 61 years) who participated in three Taijiquan sessions per week for 12 weeks. Likewise, Taijiquan was found to increase handgrip strength. In fact, the effect size of our study (within-group Cohen’s *d* = 0.80, mean age = ~64 years) is considerably larger than the effect size reported in a meta-analysis of traditional exercise training (Cohen’s *d* = 0.28, mean age = ~73 yrs), indicating the better efficacy of this Chinese conditioning practice [36]. The decline in handgrip strength with age [37] is strongly associated with old age disability [38] and cognitive impairment [39]. Thus, the present findings and their subsequent dissemination may have significant clinical implications for older persons.

Meanwhile, daily mobility is contingent upon adequate muscular strength and endurance, which becomes increasingly important with age. Tests of lower-body muscular fitness, such as the 30-second chair stand, can predict pre-clinical disability [40] in older persons. In this study, the concurrent training was found to enhance the chair stand by around two repetitions. This finding aligns with previous research that has demonstrated similar gains (around two repetitions) in either brisk walking [41] or Taijiquan practice [21]. Nevertheless, it is important to recognize the presence of the baseline effect when interpreting findings. At the onset of this study, it was seen that both groups had the ability to complete more than 17 repetitions, thereby meeting the criteria outlined in the National Physical Fitness Measurement Standards Manual (Elderly Version) for excellence in this test. The proof that the intervention group exhibited further improvement underscores the effectiveness of this training program. Therefore, it is possible that adherence to this simple and convenient training could preserve the functional capacities of the hamstring muscle group and increase the likelihood of independent living as these individuals age.

We found that 12 weeks of brisk walking and Taijiquan could enhance the sit-and-reach test by approximately 5 cm. This improvement exhibits higher effectiveness compared to the sole practice of Taijiquan (approximately 1 cm increase) [22], as well as a substantially greater advantage over other forms of concurrent exercise, such as a 2-hour training program with a 30-minute walking regimen [42]. This large effect may be due to the combination of Taijiquan and stretching exercises. In another study, Taijiquan improved functional reach more than stretching or resistance exercise alone in a group of Parkinson’s disease patients who engaged in 60-minute exercise sessions twice weekly for six months [43]. Despite the lack of clarity regarding the individual contribution of Taijiquan and stretching to the flexibility outcome, this result confirms the benefits of this concurrent training. Notably, the intervention demonstrates no effect on the back scratch. Therefore, the current training program should be modified to include additional shoulder range-of-motion activities.

Accidental falls among older persons are a global health concern. Epidemiological studies found that imbalance/dizziness accounted for one-third of older persons who fall and require emergency treatment admissions [44] and one-leg standing is the strongest predictor of individual fall risk [45]. In this regard, our study demonstrates a notable enhancement of 2.2 seconds (Hedges’ *g* = 1.07) in the one-legged standing, indicating its superiority over the practice of Taijiquan (with a mean of 1.60 seconds and a 95% confidence interval from 0.77 to 2.43 seconds, Cohen’s *d* = 0.69) [22] or engaging in walking alone (with a mean of 1.40 seconds, *p* = 0.250) [46]. It is noteworthy to emphasize that an increase of 1 second in the duration of one-legged standing has been associated with a significant 5% reduction in the risk of hip fractures [47]. This outcome holds potential for substantial healthcare benefits among older persons.

There are several non-significant findings, which should be taken seriously to note alongside the positive results, as they represent the limitations of the training program. Consequently, further refinement is necessary to improve the all-round nature of the training program for promoting healthy aging. First, 12-week brisk walking and Taijiquan did not yield any improvement in circulatory health compared to free living. Our results are compatible with two possible explanations. In the first place, neither group presented preexisting cardiovascular conditions. For example, both groups’ resting heart rates fall below the tachycardia threshold of 90 or 100 beats per minute. In contrast, 8 weeks of walking at 60–70% maximal heart rate have been shown to reduce resting heart rate and improve blood pressure regulation in tachycardic females [48]. On the other hand, Chinese people rely less on automobiles for daily commuting, and older persons walk as part of their daily living requirements [49]. As such, it is possible that cultural differences influence physical activity behaviors, rendering the 20–45 min walking redundant in terms of circulatory health for this cohort. In light of these confounding factors, additional research is required on older persons with onset cardiovascular conditions and Western residents with a higher car dependence.

Second, neither waist circumference, hip circumference, nor waist-to-hip ratio responded to this 12-week brisk walking and Taijiquan. In hindsight, the weight circumference of this sample population exceeded the Chinese-specific cutoff point for abdominal obesity [50], which may preclude our training invention from generating any meaningful biological difference without a diet intervention. A 20-year longitudinal study tracking physical activity levels and body composition during advanced aging found that body composition was unaffected by habitual physical activity in well-nourished older persons [51], indicating that exercise alone may yield contradictory long-horizon outcomes when other lifestyle factors complicate the overall issue. Meanwhile, anthropometric changes are volume [52] and intensity [53] dependent. If the objective is to improve body composition, low-intensity exercise such as walking may not be effective as expected.

Third, our result contradicts the findings of previous research indicating that walking [54] or Taijiquan [15] has positive effects on life satisfaction. If the total score on the satisfaction with life scale falls between 26 and 30, it indicates that a person is satisfied with life. In our sample population, both groups can be classified as life-satisfied at the outset, and this preexisting status may prevent the current intervention from producing further meaningful improvement. On the other hand, our sample population was healthy, requiring no assistance with daily activities, which may diminish any marginal effect from this 12-week training program. For instance, it is well documented that spinal cord injury patients who engage in regular physical activity show greater life satisfaction [55]. Therefore, the program’s effect warrants further study among older or working-age persons with daily stress, depression, and impaired daily living.

The study is subject to two practical restrictions. When designing this experiment, we made the decision to establish a control group, which was characterized by no engagement in any form of physical activity over the duration of the 12-week study. An early 2010s population-based study revealed that approximately 44.06% of middle-aged and older persons in China were classified as non-physically active [56]. Over the time, there has been a noticeable upward tendency in the proportion of physically inactive individuals among the Chinese population, as indicated by later studies conducted in 2016 (63.1% physically inactive) [57] and 2020 (67.2% physically inactive) [58]. Therefore, physical inactivity has grown increasingly prevalent in contemporary Chinese society, making our selection of a control group that does not engage in exercise ecologically valid. Strictly speaking, however, the absence of 24-hour activity monitoring jeopardizes the study’s overall validity. In addition, a dietary log was proposed as a means to validate calorie intake; however, it was not carried out. During the pilot study, it was observed that the general population cannot accurately document portion sizes, specifically within the context of Chinese food culture. Consequently, the dietary log was dropped and participants were instructed to adhere to their usual food consumption patterns for the duration of the study. The absence of precise food regulation could potentially complicate the findings with regards to circulatory health and body composition. Notwithstanding these two limitations, we encourage researchers to replicate our findings in males, older persons over 70 years of age, and non-Asian populations.

In conclusion, a 12-week concurrent brisk walking and Taijiquan improves the flexibility, muscular fitness, and balance of healthy Chinese women over 60 years old. Given that all of these physical health domains have significant clinical implications for noncommunicable diseases, accidental falls, and mortality, this study not only developed an effective type of combined exercise program but also one that is convenient and safe for older persons to perform daily. The promotion of this concurrent exercise could have long-term ramifications for China’s aging society.
